# A Case Report: Can Prioritizing Sensory Integration Therapy Help Improve Gross Motor Function in a Rare Case of Neurogenic Arthrogryposis Multiplex Congenita?

**DOI:** 10.7759/cureus.53965

**Published:** 2024-02-10

**Authors:** Nikita Sawant, Asmita Karajgi

**Affiliations:** 1 Neurophysiotherapy, The South Indian Association (SIA) College of Health Sciences, College of Physiotherapy, Thane, IND; 2 Physical Medicine and Rehabilitation, The South Indian Association (SIA) College of Health Sciences, College of Physiotherapy, Thane, IND

**Keywords:** rehabilitation, pediatric physiotherapy, neurodevelopmental technique, sensory integration therapy, arthrogryposis multiplex congenita

## Abstract

Arthrogryposis multiplex congenital (AMC) is a congenital disorder diagnosed with extremity contractures, restricted joint range of motion, foot abnormalities, and hip dislocation. The current literature emphasizes medical and surgical management, but very few studies provide insight into physiotherapy management for AMC. We reported the case of a 16-month-old male diagnosed with AMC, operated on both hips for teratologic dislocation. Physiotherapy examination was conducted, and treatment was planned based on the principles of Sensory Integration Therapy (SIT) and neurodevelopmental technique (NDT) with orthosis to assist in functional recovery. He achieved motor milestones within one year of regular physiotherapy treatment. As per our literature search, this is the first study where an attempt has been made to utilize sensory integration along with NDT for the treatment of AMC, although the clinical presentation of the patient shows more musculoskeletal abnormalities.

## Introduction

Arthrogryposis multiplex congenita (AMC) is a rare, non-progressive congenital disorder characterized by multiple joint contractures [1]. Since all the contractures are present at birth, the diagnosis of AMC is usually based on physical examination with characteristic deformities and by ultrasound in utero. Although the main problem is joint contractures, it may also be accompanied by abnormalities of other systems [2]. Typically, these children have normal intelligence and normal sensation [3]. In a few cases, prolonged physical restriction prevents environmental exploration, leading to delayed sensory development [4,5]. 

This article was previously presented as a research paper at the International Conference of Physical Therapy on January 28, 2023.

## Case presentation

Case history

**Figure 1:**
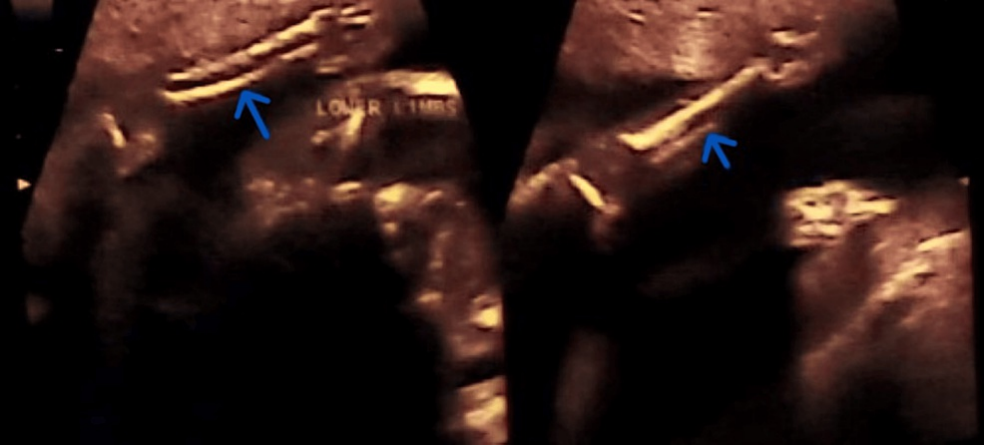
Maternal ultrasonography in the second trimester showing a fixed position of the legs with persistent knee extension (indicated by an arrow).

**Figure 2:**
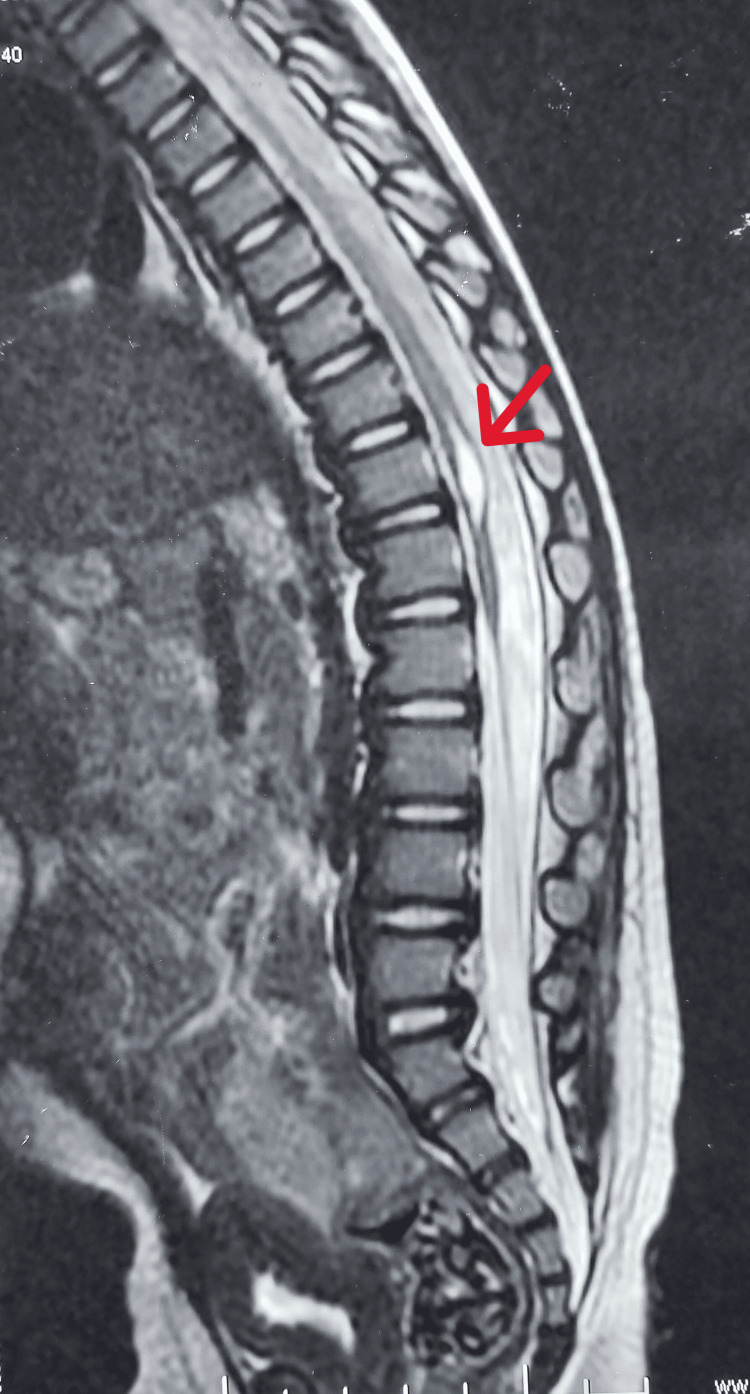
MRI of the spine showing the presence of a short segment syrinx from D10 to D12 level (indicated by an arrow).

**Figure 3:**
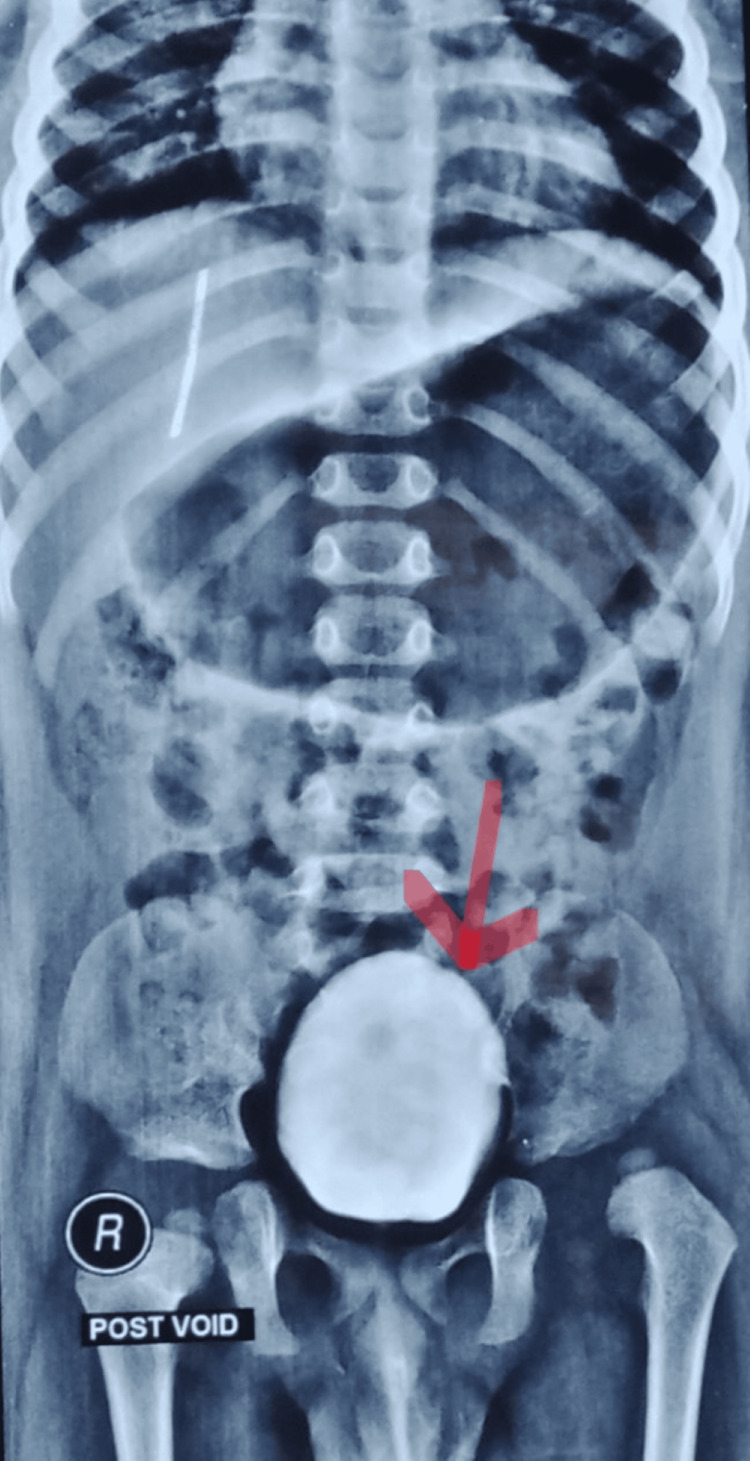
Urowater cystometry showing a large post-void volume in the urinary bladder, indicating the presence of a neurogenic bladder (indicated by an arrow).

A male child was born from a non-consanguineous marriage via emergency lower segment cesarean section at 37 weeks of gestation due to the umbilical cord wrapped around the neck. He cried immediately after birth and had no history of neonatal intensive care unit (NICU) stay. The mother is a known case of hypothyroidism and suffered from a varicella-zoster infection in the first trimester of pregnancy [1, 6,7]. He was diagnosed with AMC, including bilateral feet pes calcaneovalgus, bilateral knee extension contracture, bilateral hip teratologic dislocation, and left middle and ring finger camptodactyly. Accordingly, open reduction of the left and right hip joints was performed at the 8th and 9th months of life, respectively, followed by rectus femoris contracture release to correct lever arm dysfunction (LAD). As shown in Figure 1, maternal ultrasonography in the second trimester of pregnancy revealed a fixed position of the legs along with persistent knee extension [2]. Additionally, an MRI of the spine (Figure 2) revealed the presence of a short segment syrinx from D10 to D12 level leading to a neurogenic bladder, which was investigated by urodynamic cystometry (Figure 3) [3,8].

Clinical examination

At 16 months of age, he was referred for physiotherapy, and examination revealed that he has bilateral limited knee flexion range of motion and lower limb length discrepancy. Hip joints were diagnosed with class I LAD and ankle joints with class II LAD. The Denver II developmental screening test revealed his gross and fine motor age to be 6 months, and his language and social age to be 7 months, signifying limited environmental exploration [9]. As per the history given by the mother, tactile avoidance was suspected during daily activities, and Sensory Profile 2 confirmed that he is a ‘sensory avoider’ (avoids touch sensation more than others and performs movements much less than others). Until the examination, he had achieved gross motor milestones such as head holding, rolling on either side, prone on the forearm, and supported sitting. He skipped the creeping and crawling milestones.

Therapeutic intervention

Along with conventional physiotherapy to minimize musculoskeletal complications, Sensory Integration Therapy (SIT) and neurodevelopmental technique (NDT) were administered to achieve functional goals. According to a review article written by Warutkar VB and Krishna Kovela R, NDT is a task-oriented training that is facilitated by hands-on treatment by a physiotherapist, which enhances the patient's performance by providing purposeful and goal-oriented activity. NDT improves functional independence by activating standard postural control motions. SIT is a clinically based technique that provides play-based activities to encourage sensory processing and integration in children, improving their response to everyday stimuli [10]. The assessment was repeated every three months using Sensory Profile 2 and the Denver II developmental screening test. As written in Table 1, age-appropriate SMART (Specific, Measurable, Achievable, Reliable, and Time-bound) neurodevelopmental goals were designed to achieve the desired improvement.

**Table 1:** Age-appropriate SMART (specific, measurable, achievable, reliable, and time-bound) neurodevelopmental goals.

Treatment time period	Goal
3-month treatment goal	The child will play stacking rings with the mother in unsupported sitting using his right hand and maintain optimal alignment of head, neck, and trunk in 2 out of 4 trials, in a clinic environment.
6-month treatment goal	The child will reach for a toy kept on a lower shelf using crawling with good upper extremity and lower extremity coordination, in 3 out of 5 trials in a home environment.
9-month treatment goal	The child will scribble on the board with his right hand by performing a sit-to-stand on a height-appropriate stepper with minimum assistance in 3 out of 5 trials in a clinic environment.
12-month treatment goal	The child will play catch and throw using both hands in supported standing with optimal alignment of head, neck, trunk, and hips in neutral with knees extended in 3 out of 5 trials in a home environment. (Goal for the treatment of Lever Arm Dysfunction)

Results

After receiving treatment for almost one year, re-examinations were conducted at the age of 28 months using the Denver II developmental screening test and Sensory Profile 2. Table 2 indicates pre-treatment and post-treatment interpretations of the Denver II Developmental Screening test. Table 3 shows pre-treatment and post-treatment scores of Sensory Profile 2.

**Table 2:** Pre-treatment and post-treatment interpretation of the Denver II developmental screening test.

Test items	Pre-treatment	Post-treatment
Gross motor	6 months	17 months
Language	7 months	28 months
Fine motor - adaptive	6 months	17 months
Personal - social	7 months	28 months

**Table 3:** Indicating pre-treatment and post-treatment scores of Sensory Profile 2.

Pre -treatment				Post -treatment	
Quadrants		Raw score	Interpretation	Raw score	Interpretation
	Seeking/Seeker	15/35	Much less than others	22/35	Less than others
	Avoiding/Avoider	31/55	Much more than others	23/55	More than others
	Sensitivity/Sensor	26/65	Just like the majority of others	17/65	Just like the majority of others
	Registration/Bystander	13/55	Just like the majority of others	11/55	Just like the majority of others
Sensory and behavioral sections	General	29/50	Much more than others	11/50	Just like the majority of others
	Auditory	07/35	Just like the majority of others	07/35	Just like the majority of others
	Visual	08/30	Less than others	07/30	Less than others
	Touch	15/30	More than others	08/30	Just like the majority of others
	Movement	07/25	Much less than others	15/25	Just like the majority of others
	Oral	15/35	Just like the majority of others	10/35	Just like the majority of others
	Behavioral	08/30	Just like the majority of others	06/30	Less than others

## Discussion

As per the pathophysiology of AMC, clinical features such as multiple joint contractures and other system involvements (syrinx and neurogenic bladder) were present in the child. During childhood, exploration of the environment is necessary for the achievement of typical developmental milestones. Failure to do so will result in delayed development. In typical development, as gross motor skills develop, children are able to manipulate objects effectively in space, which provides them with a variety of sensorimotor experiences. In severe and chronic physical disabilities due to articular restrictions, limited development in gross motor skills prevents the manipulation of objects in space, subsequently affecting sensory development. Rare literature is available that focuses on the management of AMC. In the above case, a multidisciplinary approach for rehabilitation from pediatricians, pediatric orthopedic surgeons, urologists, and physiotherapists was utilized, out of which physiotherapy treatment remained the primary focus. As per a systematic review published by García Aguilar CE et al., rehabilitation primarily by physiotherapy helps to prevent the worsening of symptoms and makes the patient functionally independent [11]. As per the study presented by Lane SJ et al., sensory integration theory acts as a catalyst for development as it focuses on active, dynamic sensory-motor processes that support movement and interaction within the social and physical environment [12]. Therefore, in the first six months of treatment, the main aim was the regulation of the sensory system, which was closely monitored by Sensory Profile 2, as multisensory inputs are an integral part of everyday life and play an important role in development as it enhances the processing and interpretation of sensory events. The Denver II developmental screening test was used to monitor age-wise development [13]. For the regulation of the sensory system, activities like weight shifts on a vestibular ball, swinging, use of a rocking chair, riding a tricycle with assistance, and participation in group activities like passing the parcel, finger painting, clay molding, and use of flashcards for reading were encouraged. Once the regulation of sensory systems was achieved, the main aim was correcting LAD by increasing the hip abductor effort arm by giving stimuli through the proprioceptive system like multidirectional reachouts in weight-bearing positions and functional activities like sit-to-stand and kicking a ball. Orthoses like dynamic ankle-foot orthosis (AFO) and de-rotation belts at hip joints were used to achieve positional correction during activities. Throughout the treatment, functional activities were also carried out to improve knee range of motion, grip strength, and core muscle strength using various equipment like a Swiss ball, wobble board, aerobic stepper, and wedge. After 12 months of regular physiotherapy treatment, the child was able to sit independently, perform sit-to-stand with minimal stabilization (Video 1), cruise along the furniture, walk on a level surface with the help of orthosis and a rollator frame, and could grasp objects with his left hand, sustaining it for a few minutes. The combination of SIT and NDT has proven beneficial for the achievement of fine and gross motor development.

**Video 1:** Sit-to-stand activity on a height-appropriate stepper using bilateral ankle-foot orthoses with minimal stabilization and supervision.

## Conclusions

To conclude, prioritizing SIT and combining it with NDT, along with the support of appropriate orthoses, has proven beneficial for the gross motor, fine motor, social, and language development of a child with AMC. Although the clinical features exhibited by the child are primarily musculoskeletal, addressing sensory issues as a priority helped in improving the patient's responsiveness, leading to positive results in a shorter period. Physiotherapy management, which included a combination of SIT and NDT, enabled the child to ambulate, so he began attending school and improved his peer group interactions.
